# Patient Participation With a Mobile Phone Application for Objective Activity Assessment Before and After Spinal Fusion

**DOI:** 10.7759/cureus.10326

**Published:** 2020-09-09

**Authors:** Annelise C Sprau, Gregory Basil, Peter Borowsky, Jang W Yoon, Michael Y Wang

**Affiliations:** 1 Neurological Surgery, University of Miami Miller School of Medicine, Miami, USA; 2 Neurological Surgery, University of Pennsylvania, Philadelphia, USA

**Keywords:** mobile technology, activity tracker, spine, neurosurgery, telehealth, outcomes

## Abstract

Background

Evolution within spine surgery is driven by a surgeon’s desire for expertise and significant improvement in their patients’ quality of life. As surgeons move away from using subjective patient-reported outcome (PRO) surveys, there must be an alternative objective metric in its place. Modern iPhone (Apple Inc., Cupertino, CA) technology can be used to capture daily activity in a simple, non-user biased manner. These health data can be used to analyze objective functional status in conjunction with PRO surveys to measure surgical outcomes.

Methods

Patients who underwent an awake transforaminal lumbar interbody fusion (TLIF) between 2014 and 2018 at our institution were identified. Patients were consented and instructed to download the application “QS Access” (Quantified Self Labs, San Francisco, CA). Following data collection, we analyzed the demographic information of patients who were reached to gauge participation and feasibility of data exportation.

Results

A total of 177 patients who underwent an awake TLIF at our institution were contacted. Of those who answered, 41 (44.6%) agreed to participate and 51 (55.4%) declined to participate. When comparing those who either participated or declined, there were no significant differences in age (p=0.145), sex (p=0.589), or ethnicity (p=0.686).

Conclusion

Our pilot study examined the patient participation in the novel usage of Apple "Health" data, queried from "QS Access" (Quantified Self Labs), to objectively measure relative patient functional status surrounding spinal fusion. We demonstrated that a smartphone-based application was mostly well received by our patient cohort and has the potential to be used as an objective operative metric moving forward.

## Introduction

Advancing surgical techniques and innovative devices have revolutionized spine surgery within the past decade [1]. Many novel technologies, such as robotic spine surgery, microendoscopy-assisted procedures, and machine learning/artificial intelligence for procedural analysis, have allowed surgeons to evolve towards more minimally invasive approaches [2,3]. These advancements permit smaller incisions, less blood loss, and, ultimately, a quicker return to daily activities [4,5]. Evolution within spine surgery is not only driven by growing technical capacity, but also patients’ increasing expectations of the surgical experience [6]. Ultimately, objective surgical metrics lack value unless they reflect real and significant improvement in a patients’ quality of life. 

In order for surgeons to improve their practice in a patient-centric approach, patient experience must be measured in a reliable and reproducible fashion. Historically, this has been accomplished by patient-reported outcome (PRO) surveys, including the Oswestry Disability Index (ODI), Visual Analog Scale (VAS) back and leg pain, and the Short Form (SF) 36 Health Survey amongst others [7].

While these surveys have served as effective and easy-to-administer tools, they carry many shortcomings. Most importantly, they are highly subjective in nature and rely on patient perceptions that may not always match the clinical picture [8]. Patients may perceive an increase in functional status although objectively they are no different, and vice versa [9]. Additionally, spine surgeons as a community have yet to agree upon a single “gold-standard” PRO metric, resulting in a handful of scores used across our literature [10]. Finally, the collection and the administration of PRO surveys rely on consistent patient follow-up at pre-determined intervals to track score change over time. Without consistent follow-up for clinical care, these surveys are hardly effective in predicting surgical outcomes [11].

The use of modern technology outside of the operating room can be utilized to accurately capture daily activity in a non-user biased manner. While there is a significant body of research focused on the use of stand-alone accelerometers for this purpose, we believe that using smartphone-based accelerometer data offers a number of distinct advantages [12-14]. In this regard, we leveraged the use of a simple, free iOS iPhone application (Apple Inc., Cupertino, CA), “QS Access” (Quantified Self Labs, San Francisco, CA) for this purpose. This application is able to query Apple “Health” data, thereby yielding critical patient activity information, which include total steps, total distance traveled, and total flights climbed. These health data, in theory, can be used to analyze objective functional status supplementing subjective PRO surveys to gauge surgical outcomes. Furthermore, the phone-based application allows patients to be evaluated virtually, an aspect of particular utility in the COVID-19 era of increased telehealth utilization [15-17].

We present our single-institution, demographic analysis of patient participation in our pilot experience of the retrospective acquisition of Apple “Health” data. This initial analysis served as a way to test the feasibility of and patient comfort with a smartphone-based application, which can be used to objectively analyze pre- and postoperative functional status of patients who underwent spine surgeries.

## Materials and methods

Our initial implementation to test the feasibility of retrospective acquisition of Apple “Health” data to analyze pre- and post-operative functional status was performed solely at our institution. Inclusion criteria was all adult patients (18 years or older) who underwent elective, awake endoscopic transforaminal lumbar interbody fusion (TLIF) surgery from 2014 to 2018 by a single surgeon [18]. Patients were excluded if they did not have an iPhone, did not carry their iPhone for >75% of the day (by self-report), underwent spine surgery secondary to malignancy or trauma, are prisoners, are pregnant, or are adults unable to consent.

Patients were contacted and consented via telephone (both verbally and with an email containing written instructions) on how to download the application “QS Access” (Quantified Self Labs). “QS Access” (Quantified Self Labs) is a free iOS application for the iPhone (Apple Inc.), which extracts Apple “Health” data for the amount of time the patient has owned an iPhone (Apple Inc.). The application generates a data table of four health parameters to gauge daily energy expenditure: total steps, total distance traveled, total flights of stairs climbed, and many other surrogates of physical activities. Once the patient downloaded the application, they received written instructions on how to export their data to a secure dedicated email address. Once the data were received on the secure server, it was associated with a unique de-identified study identifier.

Demographic data were collected from the electronic medical record (EMR) and included age, sex, ethnicity, and date of surgery.

Following data collection, we primarily analyzed the demographic information of patients who were reached by telephone to gauge patient participation and feasibility of data exportation in this pilot experience. Additionally, we separated the patients who were reached into two groups: group 1 consisted of those who participated and group 2 consisted of those who did not participate. Statistical analysis with Microsoft Excel was performed using Student’s t-test or chi-squared test when appropriate, with p<0.05 denoting significance.

## Results

A total of 177 patients who underwent an awake TLIF between 2014 and 2018 at our institution were identified and contacted via the telephone by a member of the research team.

**Figure 1:**
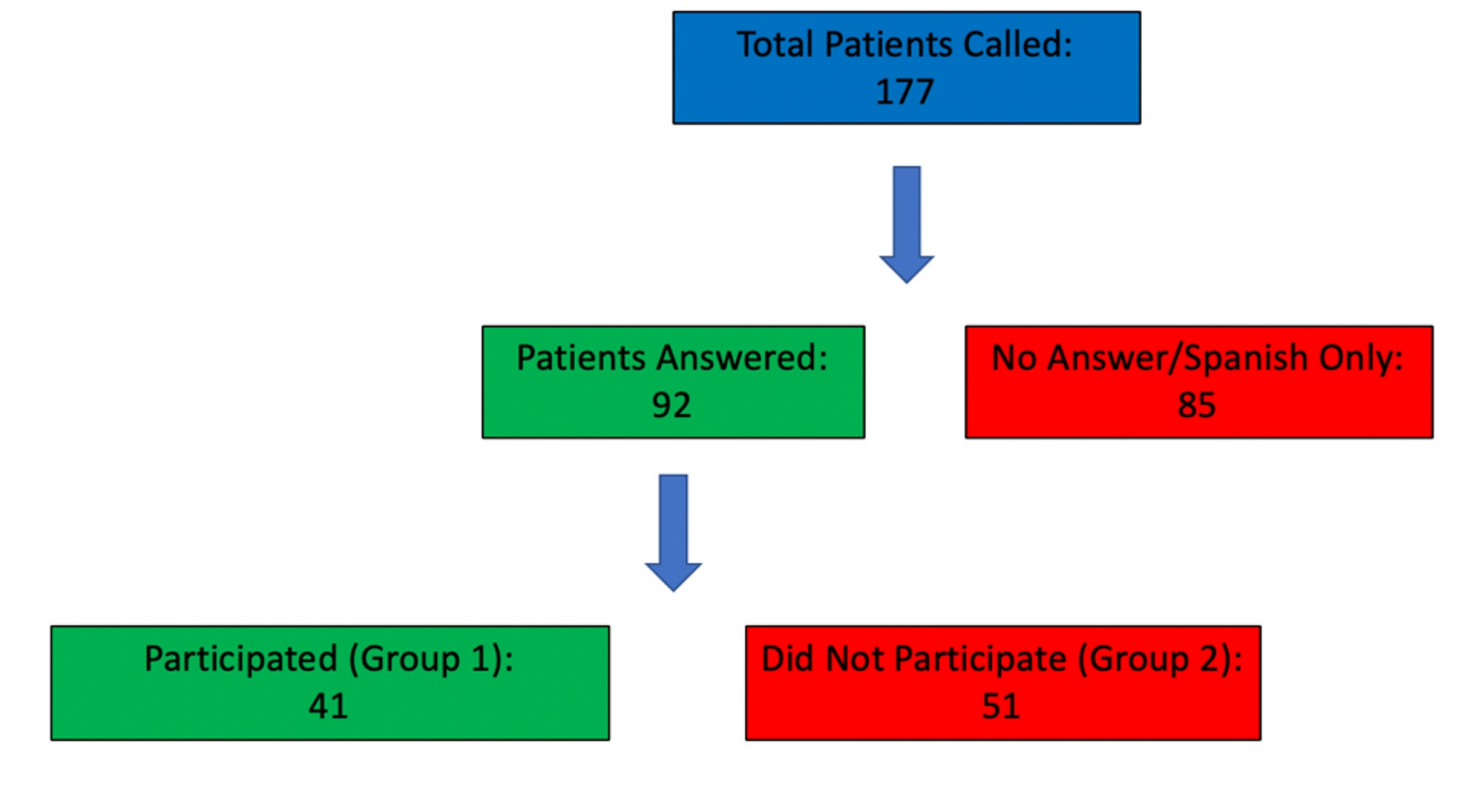
Schematic illustrating patient participation from total patient pool

Of the 177 patients, the team was able to reach 92 (52.0%) patients and was unable to reach 85 (48.0%) patients. Of the 85 patients who did not answer, 15 (17.6%) were Spanish-only speaking patients for which consent could not be effectively delivered. Of those who answered, 41 (44.6%) agreed to participate in data acquisition and 51 (55.4%) declined to participate (Figure 1, Table 1).

**Table 1:** Reasons contacted patients did not participate in the present study

Did Not Participate (n=51)	
No iPhone	43.1%
Not comfortable sharing data	19.6%
Upset with surgical outcome	15.7%
Other	15.7%
Unable to work phone	5.9%

The average age for group 1 was 64.5 ± 9.5 years, while the average age for group 2 was 67.6 ± 9.7 years. There was no significant difference in age between groups (p=0.145). Group 1 consisted of 20 (48.8%) males and 21 (51.2%) females, while group 2 consisted of 22 (43.1%) males and 29 (56.9%) females. There was no significant difference in sex between groups (p=0.589). Group 1 consisted of 12 (29.2%) patients with a self-reported Hispanic ethnicity, and 29 (70.7%) with a self-reported white ethnicity. Group 2 consisted of 13 (25.5%) patients with a self-reported Hispanic ethnicity, and 38 (74.5%) with a self-reported white ethnicity. There was no significant difference in ethnicity between groups (p=0.686) (Table 2).

**Table 2:** Demographic characteristics of 92 patients contacted in the present study SD, standard deviation

Characteristic	Value ± SD		P-value
	Group 1 (n=41)	Group 2 (n=51)	
Age (years)			
Mean	64.5 ± 9.48	67.48 ± 9.7	p=0.145
Range	46-80	47-88	
Sex (no. of patients)			p=0.589
Male	20	22	
Female	21	29	
Ethnicity (no. of patients)			p=0.686
Hispanic	12	13	
White	29	38	

## Discussion

Our pilot study examined the patient participation in the novel usage of Apple “Health" data, queried from “QS Access” (Quantified Self Labs) to objectively measure relative patient functional status surrounding lumbar spine surgery. We demonstrated that this application was generally well received by our patient population and has the potential to be used as an objective operative metric moving forward.

Patient-centered outcomes

Providers, researchers, and patient advocacy groups are shifting towards placing a higher value on patient satisfaction measures. A growing body of literature suggests that patient satisfaction can lend insight into the “true” definition of success after spine surgery [19-21]. If so, surgeons must tease out patient satisfaction in an unbiased, universal manner in order to better evolve their practices. Unfortunately, measuring patient experience is not so cut-and-dry. Patient satisfaction will necessarily be subject to a patient’s psychosocial characteristics, attitudes and beliefs, or other factors not inherently related to their surgical care [8].

Despite the inherent subjective nature of PROs, their practice has not been without surgical benefit and evolution [22]. Patient demand for cutting edge surgical techniques has pushed spine surgeons towards more appealing approaches, including minimally invasive surgery, robotic surgery, etc. This draw for technical excellence has resulted in lower postoperative morbidity, without sacrificing efficacy [23-25]. PROs have historically served as markers of surgical effect on a patient’s quality of life [26,27]. However, similar to the recent rapid shifts in surgical technique, advanced patient-centric measurement of surgical outcomes should develop toward objective metrics.

A caveat to the usage of PROs is that there is not a universally accepted pre- and postoperative metric to gauge improvement in a patient’s quality of life. A multitude of PRO surveys are currently in use, and contain questions that can vary notably between surveys [28]. Thus, it is hard to look systematically at patient outcomes across a myriad of institutions, which may prefer different means of PRO measurement. More importantly, these surveys have been traditionally administered in an outpatient setting on a face-to-face basis [29]. This requires consistent, timely follow-up in clinic, as these surveys cannot be distributed retroactively. Additionally, the challenges of the current COVID-19 pandemic greatly hinder in-person visits and have pushed many institutions to move feasible neurosurgical care towards a virtual telehealth platform [17].

Experience with mobile application 

We postulate a potential solution to the subjective PRO surveys that supports the current move towards a more virtual health experience. Our pilot experience with the application “QS Access”(Quantified Self Labs) allowed us to query health data from surgical patients as a way to measure functional status objectively surrounding surgery. The application provides a user-friendly extrapolation of health parameters that can be used to gauge energy expenditure: daily steps taken, distance walked, and flights of stairs climbed. With a carefully selected patient cohort, similar technologies can be used to create an objective measurement of meaningful surgical outcomes. Smartphone-based objective functioning has already been shown to be reliable [30], and with proper usage, can be a valid at-home outcome monitor. Additionally, the data extraction can be done in a completely virtual manner, and the application can retroactively report data, allowing surgeons to evaluate patients independent of time and place.

Due to the novelty of a smartphone-based application as a way to measure energy expenditure, the present study focused on the feasibility of patient participation and its applicability in future investigation. Thus, we chose a specific spine surgery population, performed by a single surgeon at a single institution. Our preliminary results are promising: we demonstrated that nearly 50% of patients who were reached via telephone by a member of our research team were willing to participate and share their personal Apple “Health” data. Our success with data acquisition is noteworthy, as many people may be wary of sharing personal information without face-to-face interaction. Additionally, there were no significant age, sex, or ethnic differences between those who chose to participate and those who did not, suggesting that this application can be applied universally to diverse patient populations.

Our preliminary analysis sheds light on the evolution towards a more objective and virtual measurement of meaningful outcomes from surgical intervention. Smartphone-based applications can serve as a quick and effective way to query important health information, without the tedious administration of subjective PRO surveys. With these patient-centered objective metrics, surgeons have the potential to utilize this information to craft a better overall surgical experience.

Study limitations

Our study is not without limitations. First, we were unable to successfully consent 15 Spanish-speaking patients, something that will require a secure, telehealth-driven translation modality moving forward. Additionally, data acquisition does not always ensure usability. Non-usable data could result from a blank exported file, or energy expenditure data that did not span a time frame both pre- and postoperatively. To overcome these limitations, we are planning a multi-institution study implementing this application in a broader patient sample. Additionally, this series of patients underwent surgery at a single academic center by an expert spine surgeon with the associated biases. Finally, it should be noted that while the present study examines successful enrollment/recruitment with our new platform, the full results of the study itself will be published separately in a dedicated work.

## Conclusions

Our study details the novel use of a mobile phone application to query health data to approximate daily energy expenditure from metrics, such as daily steps taken, active calories burned, distance walked, and flights of stairs climbed. We demonstrated that a smartphone-based application was generally well received by our patient population and that the process of data acquisition is feasible. We eventually plan to employ this application as a tool to measure perioperative functional status and transform the data into a predictive model of expected benefit from spinal surgical intervention based on demographic and clinical factors. 
